# Supplementary material for: A large-scale genome-wide cross-trait analysis reveals shared genetic architecture between Alzheimer’s disease and gastrointestinal tract disorders
Source: Commun Biol. 2022 Jul 18;5:691. doi: 10.1038/s42003-022-03607-2 (PMC9293965; doi:10.1038/s42003-022-03607-2)
Supplement: Supplementary file 2 — Description of Additional Supplementary Files [file 42003_2022_3607_MOESM2_ESM.pdf]

Description of Additional Supplementary Files

File name: Supplementary Data I

Description: Detailed description of GWAS datasets analysed in this study

File name: Supplementary Data 2

Description: Results showing no significant sample overlap between AD and GIT traits (based on genetic covariance intercept estimates).

File name: Supplementary Data 3

Description: Replication of genetic correlation between AD and GIT disorders.

File name: Supplementary Data 4

Description: Test for concordant SNP effects between AD (with APOE region) and GIT traits.

File name: Supplementary Data 5

Description: Replication analysis of the primary test for concordant SNP effects between AD (dataset 1) and GIT disorders (dataset 2).

File name: Supplementary Data 6

Description: Replication analysis of the primary test for concordant SNP effects between AD (dataset 2) and GIT disorders (dataset 1).

File name: Supplementary Data 7

Description: Summary of cross-traits LDSC correlation analysis between PGM and other GIT traits.

File name: Supplementary Data 8

Description: SNPs reaching genome-wide significance in the meta-analysis of AD and GERD GWAS.

File name: Supplementary Data 9

Description: Independent SNPs and loci reaching genome-wide suggestive association in the GWAS meta-analysis of AD and GERD.

File name: Supplementary Data 10

Description: Sentinel SNPs for AD associated with GIT disorders and vice versa.

File name: Supplementary Data 11

Description: SNPs and loci reaching genomewide significance in the meta-analysis of AD and PUD GWAS.

File name: Supplementary Data 12

Description: SNPs and loci replicated in the meta-analysis of AD and PUD GWAS.

File name: Supplementary Data 13

Description: Independent SNPs and loci reaching suggestive association in the metaanalysis of AD and PUD GWAS.

File name: Supplementary Data 14

Description: SNPs reaching genome-wide significance in the Meta-analyis of AD and PGM GWAS.

File name: Supplementary Data 15

Description: SNPs reaching genome-wide suggestive association for AD and PGM in the GWAS meta-analysis.

File name: Supplementary Data 16

Description: Other traits associated with 6p21.32 (near HLA-DRA).

File name: Supplementary Data 17

Description: Other traits associated with 17q21.32 (near ZNF652 and PHB).

File name: Supplementary Data 18

Description: Genomic regions shared by AD with PGM and GERD GWAS.

File name: Supplementary Data 19

Description: MR analysis results of the association between AD and GIT disorders.

File name: Supplementary Data 20

Description: Genome-wide significant genes for Alzheimer's disease.

File name: Supplementary Data 21

Description: Genome-wide significant genes for GERD GWAS.

File name: Supplementary Data 22

Description: Genome-wide significant genes for PGM.

File name: Supplementary Data 23

Description: AD and GERD shared genes reaching genome-wide significance in the FCP analysis.

File name: Supplementary Data 24

Description: Comparing Fisher's with Weighted Stouffer's method in combining pvalues for AD and GERD shared genes.

File name: Supplementary Data 25

Description: AD and PGM shared genes reaching genome-wide significance in the FCP analysis.

File name: Supplementary Data 26

Description: Significantly enriched pathways for Alzheimer's disease and gastroesophageal reflux disease.

File name: Supplementary Data 27

Description: Significantly enriched pathways for Alzheimer's disease and medicines for peptic ulcer or gastroesophageal reflux disease (PGM).

File name: Supplementary Data 28

Description: Significantly enriched pathways for GERD.

File name: Supplementary Data 29

Description: Significantly enriched pathways for AD.
